# Excitability of Upper Layer Circuits Relates to Torque Output in Humans

**DOI:** 10.3389/fnhum.2019.00359

**Published:** 2019-10-09

**Authors:** Alexander Kurz, Christian Leukel

**Affiliations:** ^1^Department of Sport Science, University of Freiburg, Freiburg, Germany; ^2^Bernstein Center Freiburg, University of Freiburg, Freiburg, Germany

**Keywords:** M1, laminar, human, TMS, nerve stimulation, motor control, force output

## Abstract

The relation between primary motor cortex (M1) activity and (muscular) force output has been studied extensively. Results from previous studies indicate that activity of a part of yet unidentified neurons in M1 are positively correlated with increased force levels. One considerable candidate causing this positive correlation could be circuits at supragranular layers. Here we tested this hypothesis and used the combination of H-reflexes with transcranial magnetic stimulation (TMS) to investigate laminar associations with force output in human subjects. Excitability of different M1 circuits were probed at movement onset and at peak torque while participants performed auxotonic contractions of the wrist with different torque levels. Only at peak torque we found a significant positive correlation between excitability of M1 circuits most likely involving neurons at supragranular layers and joint torque level. We argue that this finding may relate to the special role of upper layer circuits in integrating (force-related) afferent feedback and their connectivity with task-relevant pyramidal and also extrapyramidal pathways projecting to motoneurones in the spinal cord.

## Introduction

The motor cortex is a hub brain area for movement control in many mammalian species (Ebbesen and Brecht, 2017). Its functions, however, are little defined. A question of many previous investigations concerned the association between motor cortex activity and simple behavioral parameters, like the level of force and the movement direction. In an early investigation from Evarts (1968), he reported a positive correlation between the firing of neurons at infragranular layer 5b projecting to the spinal cord (corticospinal neurons) and the force level of the contraction. Later studies that looked at a greater number of corticospinal cells could confirm this result but emphasized the diversity of the neurons: indeed, there were some output cells at infragranular layer 5b which were well correlated with the force level, but other cells were completely uncorrelated (Cheney and Fetz, 1980; Fetz et al., 1986; Maier et al., 1993; Quallo et al., 2012). When analyzing a population of cells, the level of correlation between force output and firing rate is, in fact, lower for corticospinal neurons than what was shown for unidentified neurons in the motor cortex (Conrad et al., 1977; Hepp-Reymond et al., 1978; Wannier et al., 1991; Maier et al., 1993). This is interesting because, according to this result, the unidentified neurons coding for force could partially be corticospinal output neurons, but there must be other subtypes of neurons contained in the population response. One considerable candidate could be neurons at supragranular layers. They integrate a considerable fraction of afferent inputs (Mao et al., 2011; Hooks et al., 2013), including force-related input from the periphery, and thus force-dependent changes in afferent input may differentially affect circuits at supragranular layers in the primary motor cortex (M1).

Research focusing on the functions of circuits in different layers has been flourishing in the recent years because of its potential relevance for understanding principles of movement control (Esposito et al., 2014; Li et al., 2015; Peters et al., 2017; Arber and Costa, 2018). The purpose of the present study was to test correlations between changes in force and neuronal activity for corticospinal neurons as well as for circuits at supragranular layers in the human motor cortex. According to previous findings, we speculated that circuits at supragranular layers would show correlations with the level of force output. Estimating layer-specific activity modulations in humans is quite challenging. We recently reported a non-invasive electrophysiological method for assessing layer-specific activity modulations (Kurz et al., 2019). This method derived from a known conditioning technique involves transcranial magnetic stimulation (TMS) and spinal H-reflexes (Nielsen et al., 1993). We modified the method according to direct recordings in macaques which indicate that the first indirect (I1) descending corticospinal volley from TMS is composed of two distinct parts. The earliest part appears to originate from layer 5b neurons whereas 0.6 ms later further corticospinal neurons can be recruited into the I1 volley by inputs from layer 2/3 neurons. The high temporal resolution of the TMS conditioning technique allows to dissect the early and late part of the I1 and thereby to estimate excitability modulations of different laminar microcircuits of the human motor cortex.

In the current study, we applied this method to investigate activity modulations of laminar circuits while subjects performed auxotonic contractions with different levels of torque. Probing was performed at the movement onset and before peak torque was reached, similar to earlier studies in the monkey (Cheney and Fetz, 1980).

The main finding of the present study is a significant correlation between the excitability of M1 circuits most likely involving supragranular layer neurons and force output. No significant correlation was found for fast conducting corticospinal neurons. We presume this finding relates to the function of circuits at supragranular layers in processing task-relevant sensory feedback and the control of other infragranular layer neurons that do not belong to the pyramidal tract but are extrapyramidal and important for the control of force (Baker and Perez, 2017; Dean and Baker, 2017).

## Materials and Methods

### Subjects

Eight healthy subjects (five males and three females; 25 ± 2.8 years old) with no contraindications to TMS (Rossini et al., 2015) participated. All subjects were right-handed according to the Edinburgh Questionnaire (Oldfield, 1971) and gave written informed consent to the procedures; the study was performed in accordance with the declaration of Helsinki (latest revision in Fortaleza, Brazil) and approved by the ethics committee of the University of Freiburg, Germany (approval number 327/18).

### Electromyography

Surface electromyogram (EMG; EISA; Pfitec Biomedical Systems, Endingen, Germany) was recorded from the right flexor carpi radialis (FCR) and extensor carpi radialis muscles using bipolar surface electrodes (Blue sensor P; Ambu, Bad Nauheim, Germany) placed 2 cm apart over the muscle belly. A ground electrode was placed at the caput ulnae. Impedance was below 5 kΩ. EMG signals were pre-amplified (×100), further amplified (2×), bandpass filtered (10–1,300 Hz) and sampled at 10 kHz.

### Peripheral Nerve Stimulation (PNS)

H-reflexes were elicited with a constant current stimulator (DS7a; Digitimer, Hertfordshire, UK) by stimulating the median nerve approximately 1–3 cm proximal to the elbow joint. Stimuli consisted of square wave-pulses of 0.2 ms duration. A graphite-coated rubber pad of 5 × 5 cm was used as anode and was fixed proximal to the olecranon. A custom-made round pad (1 cm diameter) was used as the cathode and moved stepwise to detect the optimum position for eliciting H-reflexes in the FCR. The optimum was defined as the site where low stimulation intensity (5–30 mA) elicited a consistent FCR H-reflex with minimal M-wave, and no H-reflex in the antagonist extensor carpi radialis. After the optimum site was found, a self-adhesive cathode (Blue Sensor P; Ambu) was fixed at this site. An H-reflex/M-wave recruitment curve was recorded.

### TMS

Single-pulse TMS was applied over the contralateral M1 wrist area using a Magstim 2002 stimulator with a BiStim unit (Magstim, Whitland, UK) and a 50-mm figure-of-eight coil. The handle of the coil was mounted on a stand that was positioned on top of the subject chair (Manfrotto Magic Arm; Lino Manfrotto, Cassola, Italy). TMS navigation (Brainsight 2; Rogue Research, Montreal, QC, Canada) was used to monitor the position of the coil relative to the scalp to ensure that the set coil position remained the same throughout all stimuli.

The optimum site for evoking motor-evoked potentials (MEPs) was determined by a mapping procedure. The coil was held tangentially on the scalp at an angle of ~45° to the mid-sagittal plane with the handle pointing laterally and posteriorly [inducing a posterior-anterior (PA) directed current]. The motor hotspot was found by searching for the position where slightly suprathreshold PA currents produced the largest and most consistent MEP in FCR at rest.

Resting motor threshold (RMT) was determined as the minimum stimulator output (as a percentage of the maximum stimulator output) required to evoke MEPs of ≥50 μV in at least three out of five consecutive trials applied at the same intensity (Rossini et al., 1994).

### Conditioning of the H-Reflex by TMS

**Figure 1:**
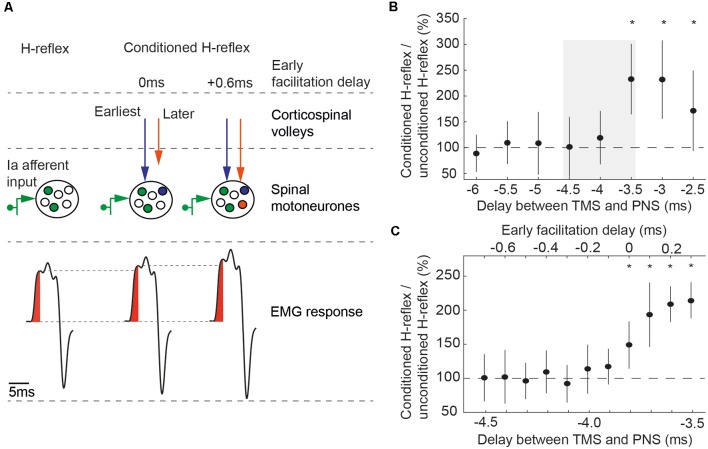
**(A)** Principle of conditioning an H-reflex evoked by peripheral nerve stimulation (PNS) with transcranial magnetic stimulation (TMS). TMS and PNS were applied together, so that TMS-triggered volleys and the afferent volleys from PNS coincided at the spinal motoneurons. This leads to an increased recruitment of spinal motoneurons (middle part) and a corresponding increase in the size of the electromyographic response in the flexor carpi radialis (FCR) H-reflex (lower part). At early facilitation delay (EFD) 0 ms, the fastest conducting corticospinal volley from TMS (blue arrow) arrive at the same time at the spinal motoneurons as the fastest conducting afferent volley from PNS. At EFD +0.6 ms, subsequent volleys (orange arrow) arrive at the same time as the fastest afferent volley. Figure adapted from Kurz et al. (2019). **(B)** EFD 0 ms was determined by a two-step procedure in each individual: we first tested delays between the application of TMS and the application of PNS from −5 ms to −2 ms, in steps of 0.5 ms (negative delays indicate that TMS was triggered after PNS). EFD 0 ms in this example was at a delay of −3.5 ms; conditioned H-reflexes at this delay and at the subsequent delays were higher than unconditioned test H-reflexes. The gray rectangle illustrates the time window tested in the second step of the procedure, shown in **(C)**. **(C)** Delays from −4.5 ms to −3.5 ms were again tested to denote EFD 0 ms in 0.1 ms steps (*P < 0.05). Figure adapted from Kurz et al. (2019).

The objective of the conditioning technique is to promote coincidence at the spinal level of TMS evoked corticospinal volleys with afferent volleys elicited by PNS (see Wiegel et al., 2018). The median nerve stimulus alone recruits a fraction of the motoneurone pool, generating an H-reflex (Figure 1A). If TMS is delivered so that the fastest descending corticospinal volley reaches the motoneurones at the same time as the afferent input, more motoneurones may be discharged, leading to a larger, facilitated H-reflex. Increasing conduction time of TMS evoked volleys allows more and more of the corticospinal volleys to influence the H-reflex. The consecutive arrival of corticospinal volleys at the spinal level lead to temporal summation, resulting in a progressive increase of the H-reflex.

In this study, additional recruitment of spinal motoneurones from two parts of the first indirect corticospinal volley (I1-volley) were studied. The first part is considered to originate from transsynaptic activation of corticospinal neurons at infragranular layer 5b (Di Lazzaro et al., 2008, 2012, 2018; Kurz et al., 2019). Supragranular layers (layer 2/3) contribute to the second part, which start 0.6 ms after the first part (Kurz et al., 2019).

A two-step procedure (rough followed by fine search) was performed to determine the time of coincident arrival of the peripheral volley and the onset of the I1-volley. In the first step (Figure 1B), delays between TMS and PNS were tested from −5 ms to −2 ms in steps of 0.5 ms, with 15 trials being recorded at each delay; in addition, 15 trials were recorded with PNS delivered alone. Note that negative delays indicate that TMS was triggered after PNS. Stimuli were delivered in 15 blocks. Each block consisted of eight trials testing all delays and an unconditioned H-reflex in a randomized order. Paired Student’s *t*-tests were used to determine the first delay (starting with the most negative delay) where the conditioned reflex was significantly different from the unconditioned reflex (*p* < 0.05); this time was taken as our initial estimate of the earliest facilitation of the H-reflex. In order to be accepted as the earliest facilitation, we required that the two subsequent less negative delays were also significantly higher than the unconditioned H-reflexes. This criterion improved the robustness of the selection procedure, making it unlikely that the earliest facilitation was wrongly determined because of outliers caused by natural variability of the electrophysiological responses. The earliest facilitation resulting from the second analysis (Figure 1C) was designated the early facilitation delay (EFD) 0 ms. EFD 0 ms (first part of the I1-volley) and EFD +0.6 ms (second part of the I1-volley) were used for testing in this study.

For all measurements, electrical stimulation intensity was adjusted to evoke H-reflexes of 15%–25% of the maximum M-wave (M_max_; Crone et al., 1990), on the upward part of the H-reflex/M-wave recruitment curve. TMS was applied with an intensity of 120% of RMT ensuring to excite neurons at different cortical layers simultaneously (Di Lazzaro et al., 2008, 2012; Di Lazzaro and Ziemann, 2013). Stimuli were applied with fixed delays of 5.5 s, to avoid post-activation depression of the H-reflex (Crone and Nielsen, 1989).

### Experimental Procedure

**Figure 2:**
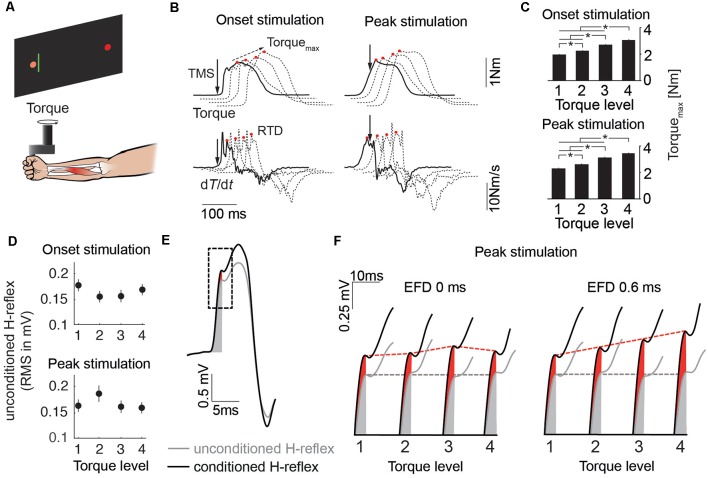
**(A)** Schematic of the experimental setup. **(B)** Mean traces of the torque signal and its first derivative for the four torque levels (solid line: Level 1, dashed lines: Level 3–4). Solid arrows indicate stimulus timing. Red circles indicate Torque_max_ and rate of torque development (RTD) respectively. Dashed arrow indicates progression of Torque_max_ over the course of the torque levels. **(C)** The bar plots illustrate mean Torque_max_ of all subjects across the four torque levels for the stimulation at movement onset and peak torque (*p < 0.05). **(D)** Unconditioned H-reflexes of all subjects (mean ± SEM) across the four torque levels for the stimulation at movement onset and peak torque. **(E)** Mean traces of the unconditioned (gray) and conditioned (black) H-reflex. Gray and red shaded area illustrate the area used to quantify H-reflex facilitation. The dashed rectangle illustrates the time window which is used in **(F)** as an expanded view for more detail. **(F)** Expanded view showing H-reflex facilitation for the stimulation at peak torque for EFD 0 ms and EFD 0.6 ms across the torque levels. Gray and red dashed lines indicate progression of unconditioned and conditioned H-reflex over the course of the torque levels.

Participants sat in front of a computer screen with their right arm resting in a fixation and the hand attached to the handle of a robotic manipulandum (Figure 2A). The computer screen provided feedback about the wrist position in the form of a cursor moving according to their wrist flexion angle from the right to the left. A trial began with the wrist in the neutral position, corresponding to the cursor placed at the right side of the screen. A visual cue (appearance of goal line) indicated to start the movement. The subject was instructed to move the cursor on a vertical line displayed at the left side of the screen. After a movement time of 600 ms, the current position of the cursor froze, and the robotic manipulandum finally pushed the wrist back to the neutral position. A new trial started with a delay of 2 s. The delay between subsequent stimuli was kept constant at 4.5 s to avoid changes in post-activation depression of the H-reflex (Crone and Nielsen, 1989).

Wrist flexions were opposed by a constant torque provided by the servomotor of the manipulandum. Four different torque levels (0.1 Nm, 0.6 Nm, 1.1 Nm, 1.6 Nm) were used (Figure 2B). Each torque level was tested in two blocks, each block comprising 60 stimulations applied in randomized order. One block consisted of 10 stimulations of conditioned H-reflexes at time intervals EFD 0 ms and EFD +0.6 ms, and one unconditioned H-reflex (30 in total), applied at two stimulation timings (at the start of the movement and just before the peak of the torque signal, solid arrows in Figure 2B). After recording of one block, a different torque level was tested. According to this protocol, a total number of 20 trials were recorded for EFD 0 ms, EFD +0.6 ms and unconditioned H-reflexes, for each torque level and each stimulation timing.

A real-time signal processing system (STIMULI; Pfitec Biomedical Systems, Endingen, Germany) was used to trigger stimulation according to online signals. The onset stimulation was triggered with a delay of 20 ms after EMG threshold (3SD above rectified EMG baseline). For the peak stimulation, the mean torque trace of the 10 test contractions prior to each block was analyzed. The PNS stimulation was set to occur approximately 100 ms prior to the peak torque.

The size of the test H-reflex was matched by using two stimulators (one for onset stimulation, one for peak torque stimulation) and by adjusting stimulation intensities (by approximately 2–8 mA) in each block.

### Data Analysis and Statistics

Data were analyzed with Matlab (Mathworks Ltd., USA, R2018a). Root-mean-square (RMS) values of the initial 0.5 ms from H-reflex onset were calculated from the unrectified FCR EMG as described in Wiegel et al. (2018). The H-reflex onset was visually determined in each subject based on the plot of superimposed unconditioned H-reflexes from all trials. Before calculating RMS values, in each trial, the offsets of the baseline EMG were corrected by setting the value at H-reflex onset to zero. Mean values were calculated for conditioned and unconditioned H-reflexes. H-reflex facilitation was calculated as the percentage change of conditioned H-reflexes compared to unconditioned test H-reflexes (conditioned H-reflex/unconditioned test H-reflex × 100%). The torque signal was low-pass filtered at 20 Hz with a zero-lag 3rd order Butterworth filter (Del Vecchio et al., 2018). The maximal torque (Torque_max_) value was determined for each trial. The maximal rate of torque development (RTD) was determined for each trial, by calculating the first derivative of the torque signal (Nielsen and Petersen, 1995). The mean Torque_max_ and RTD were calculated for each torque level. Movement time was defined as the interval between the onset of the movement (first time the movement speed exceeded 10% of its maximum) and the movement end (first time after onset the speed dropped below 10% of its maximum; D’Avella et al., 2006).

All data sets showed normality and homogeneity, tested by the Kolmogorov–Smirnov test and the Levene’s test, respectively. Torque_max_, RTD, movement time and the unconditioned H-reflexes, were analyzed by a two-way repeated-measures ANOVA with inner-subject factors STIMULATION INSTANT (ONSET and PEAK) and TORQUE LEVEL (LEVEL 1–4). Paired Student’s *t*-tests were performed for all *a priori* and *post hoc* analyses.

**Figure 3:**
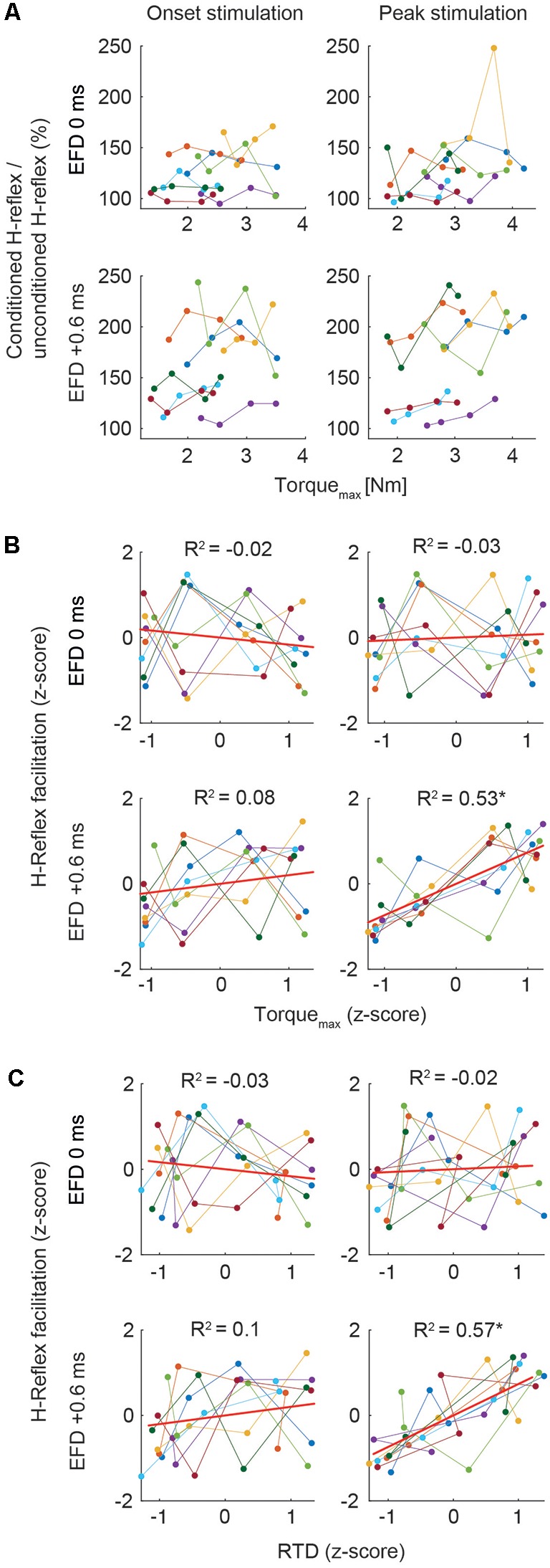
**(A)** Mean H-reflex facilitation (conditioned H-reflex/unconditioned H-reflex × 100%) and Torque_max_ (Nm) of all subjects across the four torque levels for the stimulation at movement onset and peak torque. Color code represents means of single subjects. **(B,C)** Correlations. In all graphs, *z*-scores of the four torque levels of each individual are plotted. The ordinate shows the z-score of the magnitude of H-reflex facilitation, the abscissa shows the z-score of the magnitude of Torque_max_ **(B)** and RTD **(C)**. Color code represents data of single subjects. Least squares fit for each condition is indicated with the red line. Asterisks indicate significant correlation (*p* < 0.01).

We analyzed the relation of the H-reflex facilitation and the torque data. Due to high variability of the individual baseline of H-reflex facilitation (Figure 3A), individual mean values were Fisher z-transformed to perform correlation analyses between conditioned H-reflexes and Torque_max_, and RTD, respectively. The Pearson product-moment correlation was calculated to assess the strength of bivariate correlations. Steiger’s *t*-test was used to compare bivariate correlations.

The level of significance was set to *p* < 0.05 and adjusted for multiple comparisons with Bonferroni correction. The level of significance for the correlation analyses was set to *p* < 0.01. Mean values and standard error of the mean (SEM) are reported. Data were statistically analyzed with SPSS software 24.0 (SPSS, Chicago, IL, USA).

## Results

### Torque

Torque_max_ was significantly different between torque levels in trials in which probing was performed at movement onset (Level 1: 1.97 Nm, Level 2: 2.24 Nm, Level 3: 2.72 Nm, Level 4: 3.06 Nm) and at peak torque (Level 1: 2.28 Nm, Level 2: 2. 62 Nm, Level 3: 3.12 Nm, Level 4: 3.41 Nm; Figure 2C). The ANOVA revealed a main effect for TORQUE LEVEL (*F*_(3,5)_ = 20.51, *p* < 0.01) and an interaction effect for STIMULATION INSTANT × TORQUE LEVEL (*F*_(3,5)_ = 10.12, *p* < 0.05). However, the ANOVA yielded no main effect for STIMULATION INSTANT (*F*_(1,7)_ = 0.03, *p* = 0.86).

RTD was significantly different between torque levels in trials in which probing was performed at movement onset (Level 1: 13.55 Nm/s, Level 2: 14.99 Nm/s, Level 3: 18.30 Nm/s, Level 4: 21.73 Nm/s) and at peak torque (Level 1: 14.51 Nm/s, Level 2: 15.99 Nm/s, Level 3: 18.63 Nm/s, Level 4: 21.63 Nm/s; Figure 2C). The ANOVA revealed a main effect for TORQUE LEVEL (*F*_(3,5)_ = 20.23, *p* < 0.01) and an interaction effect for STIMULATION INSTANT × TORQUE LEVEL (*F*_(3,5)_ = 10.89, *p* < 0.05). However, the ANOVA yielded no main effect for STIMULATION INSTANT (*F*_(1,7)_ = 0.02, *p* = 0.9).

### Movement Time

The movement time was not different across torque levels (Level 1: 497.36 ms, Level 2: 489.88 ms, Level 3: 475.68 ms, Level 4: 474.48 ms). The ANOVA yielded a main effect for STIMULATION INSTANT (*F*_(1,7)_ = 7.61, *p* < 0.05). However, the ANOVA revealed no main effect for TORQUE LEVEL (*F*_(3,5)_ = 3.72, *p* = 0.06) and no interaction effect for STIMULATION INSTANT × TORQUE LEVEL (*F*_(3,5)_ = 0.91, *p* = 0.48). The mean movement time for the onset stimulation was 23 ms longer than that for the peak stimulation (onset stimulation: 495 ms, peak stimulation: 472 ms), which is likely caused by the muscular twitch at the onset stimulation prolonging the movement (see top left panel in Figure 2B).

### Unconditioned H-Reflexes

The unconditioned H-reflexes were not different between the torque levels and stimulation instants (Figure 2D). The ANOVA revealed no main effect for TORQUE LEVEL (*F*_(3,5)_ = 0.57, *p* = 0.66), no main effect for STIMULATION INSTANT (*F*_(1,7)_ = 0.04, *p* = 0.85) and no interaction effect for STIMULATION INSTANT × TORQUE LEVEL (*F*_(3,5)_ = 4.38, *p* = 0.07).

### Conditioned H-Reflexes

Correlation analyses of the Torque_max_ and H-reflex facilitation at EFD 0 ms and EFD +0.6 ms yielded significant correlations only for EFD +0.6 ms when probing at peak torque (Figures 2E,F; Figures 3B,C). For the stimulation at movement onset the modulation of Torque_max_ yielded no systematic changes of the H-reflex facilitation (EFD 0 ms: *r* = −0.11, *p* = 0.55; EFD +0.6 ms: *r* = 0.34, *p* = 0.06, left panels). However, for the stimulation at peak torque EFD +0.6 ms yielded a systematic comodulation (*r* = 0.74, *p* < 0.01) with Torque_max_ (Figure 3B, bottom right panel). No such relation was present for EFD 0 ms (*r* = 0.07, *p* = 0.71). The analysis of the RTD values yielded similar results, meaning that a significant positive correlation could only be observed for EFD +0.6 ms when probing at peak torque (onset stimulation: EFD 0 ms: *r* = −0.07, *p* = 0.69; EFD +0.6 ms: *r* = 0.35, *p* = 0.05, peak stimulation: EFD 0 ms: *r* = 0.13, *p* = 0.47; EFD +0.6 ms: *r* = 0.76, *p* < 0.01). Steiger’s *t*-tests yielded no significant differences between correlations of Torque_max_ and H-reflex facilitation vs. RTD and H-reflex facilitation (onset stimulation: EFD 0 ms: *Z* = 0.191, *p* = 0.85; EFD +0.6 ms: *Z* = 0.105, *p* = 0.92; peak stimulation: EFD 0 ms: *Z* = −0.405, *p* = 0.69; EFD +0.6 ms: *Z* = 0.323, *p* = 0.75).

## Discussion

This study aimed at investigating the relation of layer-specific activity of microcircuits in human M1 and changes in joint torque. Therefore, modulations of the early and late component of the I1-volley (EFD 0 ms and EFD +0.6 ms) were tested with a non-invasive electrophysiological technique involving TMS and H-reflexes (Kurz et al., 2019). According to Kurz et al. (2019), the early component of the I1-volley contains contributions from fast conducting corticospinal neurons in layer 5b. The late component contains also contributions from circuits at supragranular layers. We applied the same experimental protocol as in the original study by Kurz et al. (2019). When probing at peak torque in the current study we found a strong correlation of the late component with different torque levels. There was no such correlation for the early component of the I1-volley. As unconditioned H-reflexes were matched across testing conditions we conclude that circuits at supragranular layers but not the tested population of corticospinal neurons were associated with changes in torque output.

It may seem intuitive to think that output cells of the motor cortex, which are strongly influencing the activity of spinal motoneurones, are associated with the level of force. However, as it was briefly mentioned in the introduction of this article, results from a series of studies (Cheney and Fetz, 1980; Fetz et al., 1986, 1989; Maier et al., 1993) indicate a more complex relationship of the firing of these neurons and torque output. Some neurons indeed show this relationship, but others do not or even the reverse, i.e., a lower firing rate with increased force levels. Muir and Lemon (1983) analyzed the activity of corticospinal cells in M1 in monkeys performing a power grip (comparable to the task used in this study) and a precision grip, requiring fractionated use of the digits. The activity of the recorded corticospinal cells was modulated only for the precision grip, but not the power grip. This indicates that the activity of these cells was particularly associated with the control of independent finger movements, but not with changes in force. It is important to mention that TMS in our study is probing the excitability of a large number of output neurons. With this method, the activity of the population response can be estimated, but not the behavior of single neurons. Our finding that concern excitability modulations at EFD 0 ms is consequently in support of earlier observations, because it indicates that the population of corticospinal neurons tested at EFD 0 ms showed no systematic modulation in its activity with altered torque output.

Why was there only a correlation between EFD +0.6 ms tested at peak torque and the torque level? We have no clear answer to that question but provide potential explanations. It is known that a large part of peripheral afferent input from receptors signaling changes in force (Ib afferents) and muscle length (Ia afferents) target layer 2/3 circuits in the motor cortex (Mao et al., 2011; Hooks et al., 2013). Some of the circuits at these supragranular layers that were assessed with our method may integrate the afferent signals. A variation of afferent input to these circuits based on the force level may be reflected in terms of a changed neural activity. The changes in neural activity would likely be measurable when probing at peak torque but not movement onset. This may relate to the timing of afferent feedback arriving at the motor cortex. Proprioceptive input from a muscular contraction will take around 50 ms to reach cortical circuits (Fetz et al., 1980), which exceeds the time point of probing at movement onset by roughly 20 ms (probing was performed roughly 30 ms after movement onset). In contrast to that, at peak torque, afferent feedback has far reached the motor cortex (Rosén and Asanuma, 1972; Wong et al., 1978; Cheney and Fetz, 1984; Koželj and Baker, 2014).

Circuits at supragranular layers can activate many different descending pathways. They connect to different output layers, 5 and 6 (Weiler et al., 2008). The processing of potentially task-relevant sensory feedback (see previous paragraph) and the connectivity of upper layer circuits not only with pyramidal but also extrapyramidal pathways promote upper layer circuits for force control. Upper layer circuits connect to output neurons in M1 which have connections to neurons in the reticular formation of the brainstem (Kably and Drew, 1998; Matsuyama et al., 2004), giving rise to the reticulospinal tract as one major descending extrapyramidal pathway (Kuypers and Brooks, 1981; Lemon, 2008). The reticulospinal tract has indeed been shown to contribute to contractions that require larger force output, such as the power grip (Baker and Perez, 2017).

The correlation was found between EFD +0.6 ms and Torque_max_, but also between EFD +0.6 ms and the first derivative of the torque signal indicating the maximum torque change (RTD). As shown in Figure 2B, the maximum torque change occurred clearly before the probing instant at peak torque. This means that RTD was a true estimate of the motor output, unbiased from the twitch induced from TMS. This is in contrast to the variable Torque_max_, which in terms of timing occurred after the stimulation was performed, and could thus be biased by the induced twitch. It is well known that RTD is closely related to the maximum magnitude of exerted torque (Folland et al., 2014). This means that the correlation between EFD +0.6 ms and Torque_max_ was unlikely caused by the induced twitch torque from TMS. EFD +0.6 ms was slightly better correlated to the RTD than to Torque_max_, but this difference was not significant.

Although the present results indicate a significant relationship between activity changes of supragranular circuits and changes in torque output, we repeatedly emphasize the non-invasive nature of the methods as a limiting factor. We already discussed (Kurz et al., 2019) that the use of a non-invasive method makes definite claims about neuronal mechanisms impossible. Specifically, TMS in the present study was likely targeting M1, and because of our knowledge about how it affects cortical neurons (Di Lazzaro et al., 2008, 2012, 2018; Di Lazzaro and Ziemann, 2013), and by adding PNS, we propose to have dissected activity at supra- and infragranular layers. However, there might be a chance that TMS was in fact not targeting M1, and thus that the results which we report point to other mechanisms, e.g., connections between the premotor cortex and the motor cortex (see also Kurz et al., 2019).

In summary, in the present study, we show correlations between excitability changes of the later part of the I1-volley from TMS and changes in torque output. There was no such correlation between excitability modulations of the first part of the I1-volley and changes in torque output. According to our knowledge about the origin of the parts of the I1-volley and experiments in non-human primates about force control we conclude that upper layer circuits in the human motor cortex are stronger associated with changes in torque than fast conducting corticospinal neurons at layer 5b. We propose that (task-relevant) sensory input at supragranular layers may be the reason for the excitability changes we observed. These findings aim to improve our understanding of how torque is controlled by neural circuits of the human brain.

## Data Availability Statement

All datasets generated for this study are included in the manuscript.

## Ethics Statement

All subjects gave written informed consent to the procedures; the study was performed in accordance with the declaration of Helsinki (latest revision in Fortaleza, Brazil) and approved by the local ethics committee in Freiburg (approval number 327/18).

## Author Contributions

AK and CL conceived and designed the research, drafted the manuscript, edited and revised the manuscript, prepared figures and interpreted results of experiments. AK performed the experiments and analyzed the data. All authors approved the final version of manuscript.

## Conflict of Interest

The authors declare that the research was conducted in the absence of any commercial or financial relationships that could be construed as a potential conflict of interest.
